# Differential expression of topoisomerase I and RAD52 protein in yeast reveals new facets of the mechanism of action of bisdioxopiperazine compounds

**DOI:** 10.1038/sj.bjc.6690767

**Published:** 1999-11

**Authors:** B van Hille, X Clerc, A M Creighton, B T Hill

**Affiliations:** Division de Cancérologie, Centre de Recherche Pierre Fabre, 17 av. Jean Moulin, Castres Cédex 81106, France; Medicinal Chemistry Laboratory, Department of Reproductive Physiology, St Bartholomew’s Hospital Medical College, 51–53, Bartholomew Close, London ECIA 7BE, UK

**Keywords:** human, yeast, topoisomerase I, RAD52, DNA repair, bisdioxopiperazine

## Abstract

A screening procedure which permits identification of compounds based on their activities against specific biological targets directly in a living organism, *Saccharomyces cerevisiae*, has been established as part of our new drug discovery programme. Use of this assay has provided the first direct evidence that TOP1 and RAD52 proteins are involved in the mode of action of bisdioxopiperazine ICRF compounds, which thus express a mode of action quite distinctive from the other known TOP2 inhibitors evaluated. The functional assay is based on a comparison of pairs of yeast differing in their phenotypes by specific traits: the expression or lack of expression of ectopic human DNA topoisomerase I, with or without that of the *RAD52* gene. Amongst a series of anticancer agents, inhibitors of topoisomerase I (camptothecin) were identified as such in yeast expressing human topoisomerase I, whilst the presence or absence of RAD52 protein permitted the discrimination of compounds generating double-stranded DNA breaks, either directly (bleomycin) or involving DNA adduct formation (cisplatin), or indirectly with DNA damage mediated via inhibition of the topoisomerase II enzyme (etoposide). Notably, however, both the RAD52 protein and the lack of TOP1 enzyme appeared implicated in the cytotoxic activities of the series of bisdioxopiperazine ICRF compounds tested. This functional assay in a living organism therefore appears to provide a valuable tool for probing distinctive and specific mode(s) of action of diverse anticancer agents. © 1999 Cancer Research Campaign


*British Journal of Cancer* (1999) **81**(5), 800–807
© 1999 Cancer Research Campaign
Article no. bjoc.1999.0767

### Differential expression of topoisomerase I and RAD52 protein in yeast reveals new facets of the mechanism of action of bisdioxopiperazine compounds

**B van Hille[1], X Clerc[1], AM Creighton[2] and BT Hill[1]**

[1]Division de Cancérologie, Centre de Recherche Pierre Fabre, 17 av. Jean Moulin, 81106 Castres Cédex, France; [2]Medicinal Chemistry Laboratory, Department of Reproductive Physiology, St Bartholomew's Hospital Medical College, 51–53, Bartholomew Close, London ECIA 7BE, UK

**Summary** A screening procedure which permits identification of compounds based on their activities against specific biological targets directly in a living organism, *Saccharomyces cerevisiae*, has been established as part of our new drug discovery programme. Use of this assay has provided the first direct evidence that TOP1 and RAD52 proteins are involved in the mode of action of bisdioxopiperazine ICRF compounds, which thus express a mode of action quite distinctive from the other known TOP2 inhibitors evaluated. The functional assay is based on a comparison of pairs of yeast differing in their phenotypes by specific traits: the expression or lack of expression of ectopic human DNA topoisomerase I, with or without that of the *RAD52* gene. Amongst a series of anticancer agents, inhibitors of topoisomerase I (camptothecin) were identified as such in yeast expressing human topoisomerase I, whilst the presence or absence of RAD52 protein permitted the discrimination of compounds generating double-stranded DNA breaks, either directly (bleomycin) or involving DNA adduct formation (cisplatin), or indirectly with DNA damage mediated via inhibition of the topoisomerase II enzyme (etoposide). Notably, however, both the RAD52 protein and the lack of TOP1 enzyme appeared implicated in the cytotoxic activities of the series of bisdioxopiperazine ICRF compounds tested. This functional assay in a living organism therefore appears to provide a valuable tool for probing distinctive and specific mode(s) of action of diverse anticancer agents. © 1999 Cancer Research Campaign

**Keywords:** human; yeast; topoisomerase I; RAD52; DNA repair; bisdioxopiperazine

Clinically useful anti-tumour agents have widely differing biochemical and molecular mechanisms of action, depending of their target(s) among the cellular components, notably the metabolism of DNA (Pinedo et al, 1997). In this respect, DNA topoisomerases and proteins involved in DNA repair are of particular interest. DNA topoisomerases I (TOP1) and II (TOP2) are essential enzymes differentially regulating many aspects of the topology of nucleic acids, including DNA replication, transcription, chromosome structure, condensation and segregation, as well as the organization of the nuclear matrix (Wang, 1985; Osheroff, 1989; Chen and Liu, 1994). Among several mechanisms, some of the topoisomerase-targeting anti-tumour agents exert their cytotoxic effects by stabilizing cleavable complexes, an otherwise transitory step of the catalytic cycle of the topoisomerase enzyme (Chen and Liu, 1994). From this observation, it has been proposed that this stabilization creates persistent DNA strand breaks and, therefore, that cells are killed because the enzyme is converted into a 'poison' or a DNA damaging agent, and not just merely from a lack of enzyme activity (Corbett and Osheroff, 1993; Chen and Liu, 1994; Pommier et al, 1994). The pathways committed in the repair of DNA strand breaks generated by the inhibition of topoisomerase, as described above, or in response to various classes of DNA damaging agents are now gradually being deciphered (Chaney and Sancar, 1996; Wood, 1996; Barret and Hill, 1998). Notably, extensive studies of the RAD52 epistasis group in yeast have already permitted a better understanding of the molecular mechanism(s) of recombinational repair of double-stranded DNA breaks (Friedberg et al, 1991; Hays et al, 1995). One of the best characterized genes in this group is *RAD52* (Milne and Weaver, 1993), whose inactivation leads to a deficiency in recombination and double-stranded DNA break repair. Persistent unresolved double-strand breaks destabilize DNA and can lead to $G_2$ arrest and then, as a consequence, lethality (Bennett et al, 1997).

The possible intricate relationships existing between the mechanisms of action of topoisomerases, DNA metabolism and the DNA repair pathways revolving around the common DNA substrate therefore suggested that the mode of action of drugs might be best investigated in a multifactorial model in order to dissect their potentially differential mechanism(s) of action.

In this study, we have developed a functional assay focusing on specific molecular pathways of potential importance in the field of chemotherapy such as those associated with the topoisomerases and double-stranded DNA repair. Specifically, the assay is based on analyses of the differential sensitivities of pairs of yeast with phenotypes differing by only specific traits, namely expression or not of either the RAD52 protein, or of the ectopic human TOP1 enzyme. Following its validation using a series of standard anti-tumour agents, including known TOP1 and TOP2 inhibitors, as well as DNA-damaging agents, this assay was used as a tool to provide a better understanding of the mode of action of a series of bisdioxopiperazines, ICRF compounds (Andoh and Ishida, 1998), certain of which have been described as inhibitors of the catalytic

*Received 23 February 1999*
*Revised 6 May 1999*
*Accepted 7 May 1999*

*Correspondence to:* BT Hill

**Table 1** Characteristics of the yeast transformants used in these studies

| Yeast transformant | Genotype/plasmid host | Phenotypic status | | |
|---|---|---|---|---|
| | | yTOP1 | hTOP1 | RAD52 |
| JN394 | *MATa ura3–52 leu2 trp1 ade1–2 his7 ISE2 top1::TRP RAD52::LEU2* | – | – | – |
| JN362a | *MATa ura3–52 leu2 trp1 ade1–2 his7 ISE2 top1::LEU2* | – | – | + |
| Y(+/–) | as JN394 + pYX112–hTOP1B | – | + | – |
| Y(–/–) | as JN394 + pYX112 | – | – | – |
| Y(–/+) | as JN362a + pYX112 + pRS414 | – | – | + |
| Y(+/+) | as JN362a + pYX112-hTOP1B + pRS414 | – | + | + |

activity of TOP2 through trapping of the cleavable complexes, after the religation of cleaved DNA ends (Roca et al, 1994; Sehested and Jensen 1996; van Hille and Hill, 1998).

### MATERIALS AND METHODS

### Chemicals and drugs

Amsacrine, cisplatin and doxorubicin were purchased from Sigma (Saint-Quentin Fallavier, France), camptothecin from Cipla Ltd (Bombay, India), mitoxantrone from Lederle (Paris-La Défense, France), bleomycin from Roger Bellon (Neuilly-sur-Seine, France), ICRF-187 (dexrazoxane) from Chiron (Suresnes, France) and fungizone from Gibco-BRL (Gaithersburg, MD, USA). The other test compounds, i.e. 10-hydroxy-camptothecin, etoposide, ICRF-159 (razoxane), TOP 53 and vinorelbine were provided by Pierre-Fabre Médicament (Castres, France). ICRF-201, ICRF-202, ICRF-186 and ICRF-193 were synthesized essentially as described in the published literature (Creighton, 1971, 1974). Test compounds were dissolved in 10% dimethylsulphoxide (DMSO) obtained from Sigma (Saint-Quentin Fallavier, France), except for cisplatin which was dissolved in 0.9% sodium chloride and, with bleomycin, a $Fe^{2+}(NH_4)_2$ solution was used.

### Yeast strains, plasmids and construction of plasmids

The yeast strains JN394 (*MATa ura3–52 leu2 trp1 ade1–2 his7 ISE2 top1::TRP RAD52::LEU2*) and JN362a (*MATa ura3–52 leu2 trp1 ade1–2 his7 ISE2 top1::LEU2*) and the centromeric plasmid construct pYX112-hTOP1B that allows for the overexpression of human TOP1 under the control of the promoter TPI, were kindly provided by Dr JL Nitiss (St Jude Children's Research Hospital, Memphis, TN, USA).

Strain JN394 was transformed with plasmid construct pYX112-hTOP1B and subclones designated Y(+/–) were selected. Similarly, subclones of yeast JN394 transformed with a plasmid pYX112 bearing only the selective URA3 gene were also selected and designated Y(–/–). Yeast JN362a was transformed with plasmids pYX112 and pRS414 so as to select a strain Y(–/+) regaining prototrophy for, respectively, uracil and tryptophan. JN362a was also successively transformed with the plasmids pYX112-hTOP1B and pRS414, generating the yeast transformant Y(+/+), so as to share the same growth requirements as the three previously selected yeast recombinants, namely, Y(+/–), Y(–/–) and Y(–/+). These four recombinants therefore differed from each other on the basis of the absence or overexpression of hTOP1 and/or absence or normal expression level of the RAD52 protein. For clarity in the text, yeast recombinants developed in this study were designated according to their phenotypic characteristic, i.e. symbol in brackets correspond to expression (+) or not (–) of TOP1 enzyme (left symbol) and RAD52 protein (right symbol). The construction of yeast used in this study, along with their genotypic and phenotypic characteristics is detailed in Table 1.

### In vivo drug screening assays

Yeast strains were stored, propagated and grown in selective minimal media, as described earlier (Ausubel et al, 1995). Yeasts were transformed with an electroporator (Subra model GHT 1287B, Toulouse, France) at 625 V for one pulse of 16 ms. The assay was performed as described previously (van Hille and Hill, 1998). Briefly, yeast strains in exponential-growth phase were adjusted to $10^7$ cell $ml^{-1}$. Thereafter, 2 µl aliquots from serial tenfold dilutions of each culture were seeded onto agar plates containing selective minimal media and a final concentration of 1% DMSO with or without test compound, at various concentrations. Plates were incubated at 30°C for 4 days. The surface of all the plates was then digitally processed using a 'Geldoc 1000' fluorescent gel documentation system (Bio-Rad, Hercules, CA, USA) and the density of yeast growth for each inoculum was quantitated with the associated Molecular Analyst software provided.

### Measurement of drug sensitivities

The effects of each drug concentration on the survival of each individual yeast recombinant was determined as a percentage of the growth observed on the control plate to which only DMSO had been added. Starting with values obtained from inoculi from stock cultures of each yeast strain, the GraphPad Prism software was used to calculate and draw non-linear regression curves of cytotoxicity, with the 'sigmoïdal dose–response (variable slope)' mode. Regression curves for each strain were superimposed on the same graph. The top plateau corresponds to 100% growth relative to the control and the bottom one to 100% cytotoxicity. Concentrations of each compound which reduced the density of growth by 50%, $IC_{50}$ values, were also calculated and used as a measure of the cytotoxicity of each compound against an individual yeast strain. Additionally, results from the serial dilutions of yeast on the plates were used visually to confirm the effect of the compound on the viability of the yeast. All results were reproducible in at least two independent experiments. Furthermore, each experiment included as controls, camptothecin and etoposide, to check for the maintenance of, respectively, the variation in TOP1 and RAD52 expressions, through their respective differential sensitivities.

© 1999 Cancer Research Campaign 

**Table 2** Differential effects of a series of antitumour agents on the growth of yeast transformants differing in terms of their hTOP1 and/or RAD52 status

| | Y(+/–) $IC_{50}$ | Y(–/–) $IC_{50}$ | Y(–/+) $IC_{50}$ | Y(+/+) $IC_{50}$ | RAD52 – TOP1 –/+ Y(–/+)/Y(+/+) Ratio R1[a] | RAD52 + TOP1 –/+ Y(–/–)/Y(+/–) Ratio R2[b] | TOP1 – RAD52 –/+ Y(–/–)/Y (–/+) Ratio R3[c] | TOP1 + RAD52 –/+ Y(+/–)/Y(+/+) Ratio R4[d] | Constant trait Variable trait |
|---|---|---|---|---|---|---|---|---|---|
| Group 1 test compound | | | | | | | | | |
| Camptothecin | 0.12 | > 10 | >10 | 0.13 | >77 | > 83 | NE | 0.9 | |
| 10-Hydroxycamptothecin | 1.8 | > 100 | >100 | 1.4 | >71 | > 55 | NE | 1.3 | |
| Group II test compound | | | | | | | | | |
| Etoposide | 3.3 | 2.9 | > 100 | > 100 | NE | 0.9 | < 0.1 | < 0.1 | |
| Amsacrine | 52 | 40 | > 100 | > 100 | NE | 0.8 | < 0.4 | < 0.5 | |
| Mitoxantrone | 4.6 | 6.7 | > 100 | > 100 | NE | 1.4 | < 0.1 | < 0.5 | |
| TOP 53 | 1.1 | 1.1 | > 100 | > 100 | NE | 1.0 | <0.1 | < 0.1 | |
| Bleomycin | 5.8 | 7.9 | 15 | 15 | 1.0 | 1.4 | 0.5 | 0.4 | |
| Cisplatin | 4.0 | 3.2 | 27 | 30 | 0.8 | 1.3 | 0.1 | 0.1 | |
| Doxorubicin | 1.1 | 1.1 | 9.8 | 9.1 | 1.1 | 1.0 | 0.1 | 0.1 | |
| Group III test compound | | | | | | | | | |
| ICRF-159 | 100 | 18 | 57 | > 200 | < 0.4 | 0.2 | 0.3 | < 0.5 | |
| ICRF-186 | 29 | 6.8 | 17 | > 100 | < 0.2 | 0.2 | 0.4 | < 0.3 | |
| ICRF-187 | > 200 | 100 | > 200 | > 200 | NE | NE | NE | NE | |
| ICRF-193 | 100 | 3.5 | 28 | > 100 | < 0.3 | 0.4 | 0.1 | < 1 | |
| ICRF-201 | > 100 | 42 | > 100 | > 100 | NE | NE | NE | NE | |
| ICRF-202 | 15 | 5.6 | 17 | > 100 | < 0.2 | 0.4 | 0.3 | <0.2 | |

[a]Ratio R1 = [$IC_{50}$ from Y(–/+)]/[$IC_{50}$ from Y(+/+)]; [b]ratio R2 = [$IC_{50}$ from Y(–/–)]/[$IC_{50}$ from Y(+/–)]; [c]ratio R3 = [$IC_{50}$ from Y(–/–)]/[$IC_{50}$ from Y(–/+)]; [d]ratio R4 = [$IC_{50}$ from Y(+/–)]/[$IC_{50}$ from Y(+/+)]. $IC_{50}$ values, in µM, correspond to drug concentrations that reduced the density of growth by 50%. Drug-induced differential growth inhibition between two yeast recombinants differing by only one trait were assessed as the ratios R1, R2, R3 or R4. Depending on the ratio values obtained, three groupings of drugs were defined: group I those with ratios R1 and R2 above 1.3; group II those with ratios R3 and R4 below 0.7, and group III those not falling in previous two groups. NE = non evaluable.

Comparison of the relative differences in sensitivities to a given compound expressed by two yeast recombinants is based on previous work examining cross-resistance in mammalian cells (Shen et al, 1995) and yeast (van Hille and Hill, 1998). Briefly, the respective drug-sensitivity of the four yeast transformants are compared two-by-two, depending on the constant and variable parameters via four different ratios R1 = [$IC_{50}$ from Y(–/+)]/[$IC_{50}$ from Y(+/+)], R2 = [$IC_{50}$ from Y(–/–)]/[$IC_{50}$ from Y (+/–)], R3 = [$IC_{50}$ from Y (–/–)]/[$IC_{50}$ from Y(–/+)] and R4 = [$IC_{50}$ from Y(+/–)]/[$IC_{50}$ from Y(+/+)] (Table 2). Furthermore, as demonstrated earlier (van Hille and Hill, 1998) and confirmed in this study (Figure 1), standard deviations did not exceed 30%. Therefore, different sensitivities between yeasts were considered significant when ratios were either below 0.7 or above 1.3 (Table 2).

### RESULTS

### Validation of the functional assay in yeast

The four yeast recombinants developed in this study, whose phenotypic characteristics are summarized in Figure 1A, had similar growth rates in identical media, and no deleterious effect on their growth was shown to be exerted by up to 1% DMSO in the agar medium (data not shown). Furthermore, following digital processing of the surface of each agar plate, the density of yeast growth issuing from each inoculum was quantitated and translated into curves of growth inhibition as a function of drug concentration. Representative data for three of the antitumour agents evaluated are illustrated in Figure 1B–D. The extent of the standard deviations relating to each data point are illustrated for only one of these test compounds, etoposide (Figure 1C), for the sake of clarity. These values rarely exceeded 30%, possibly reflecting the cumulative variations inherent in the preparation of stock solution and dilutions of compounds tested, as well as the setting of agar plates, the deposition of inoculi on these plates and of the growth of the yeasts themselves (van Hille and Hill, 1998). Overall, the $IC_{50}$ values recorded for the different compounds tested varied over more than two logs ranging from 0.1 to 50 µM (Table 2). It is difficult to attribute any definite significance to these numbers, since these $IC_{50}$ values merely reflect the level of growth inhibition induced by each test compound whose bioavailability may in fact be limited due to: (i) the permeability barrier at the cell wall of the yeast; this permeability appears to vary from drug to drug, as previously described (Nitiss and Wang, 1988), and (ii) the half-life of each test compound in the agar medium. Therefore, $IC_{50}$ values measured using yeast transformants cannot readily be compared directly with data obtained using mammalian cells, devoid of this cell wall. However, according to similarities in the drug-induced patterns of differential growth inhibition identified from the ratios R1, R2, R3 and R4 obtained with this assay, the anti-tumour compounds tested could be classified into three separate groups (Table 2).

### Identification of inhibitors of TOP1 enzyme

Two known inhibitors of TOP1, camptothecin (Wall et al, 1966) and 10-hydroxy-camptothecin (Kingsbury et al, 1991), were tested in this in vivo functional assay. Both compounds induced a characteristic pattern of differential growth inhibition amongst the four yeast recombinants tested, as exemplified by the curves of growth inhibition generated by camptothecin (Figure 1B), clearly associated with the increased expression of TOP1. These data essentially confirm previous reports (Eng et al, 1988, Bjornsti et al, 1988). Indeed, the growth of yeast Y(–/–) and Y(–/+) was not different from that measured on a control plate free of test compound,

 *© 1999 Cancer Research Campaign*

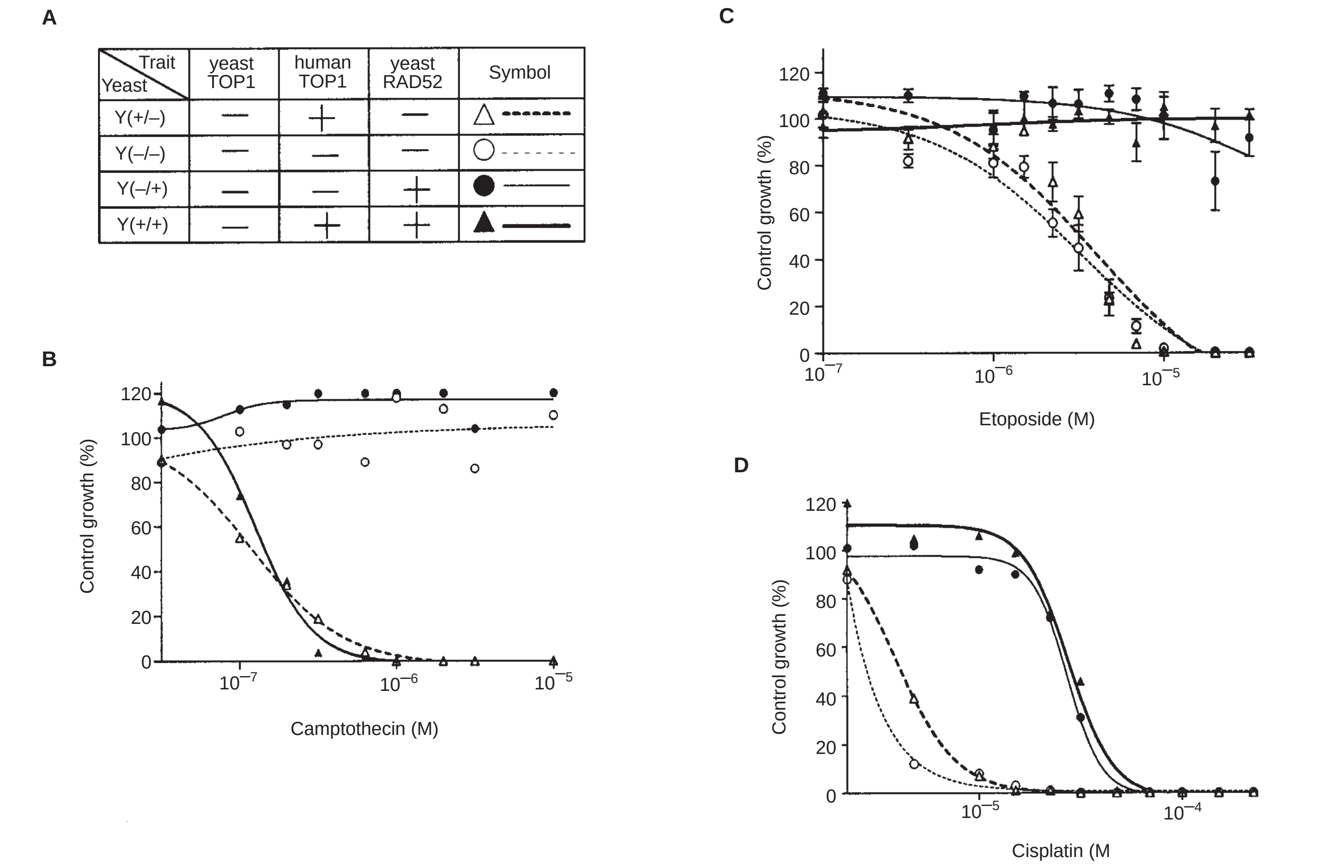

**Figure 1** Curves of growth inhibition. Yeast transformants Y(+/–) : (△ ----); Y(–/–): (○ ----); Y(–/+) : (● —) and Y(+/+) : (▲ —), whose respective phenotypic characteristic and symbols used for constructing the curves are summarized in panel **A**, were challenged with camptothecin (**B**), etoposide (**C**) or cisplatin (**D**). Data were plotted using a Windows-based Prism program whereas the growth of yeast Y(+/–) and Y(+/+) was severely impaired at camptothecin concentrations lower than 0.1 μM and these growth inhibition curves were superimposable (Figure 1B). Corresponding $IC_{50}$ values were, respectively, 0.12 μM and 0.13 μM (Table 2, group I). The RAD52-independent influence of the TOP1 status on the sensitivity of a pair of yeasts challenged with camptothecin could be identified by examining the two ratios of the $IC_{50}$ values obtained, from pairs of yeasts exhibiting either the wild-type *RAD52* gene, i.e. R1 or a 'knocked-out' *RAD52*Δ gene, i.e. R2. Values of these ratios R1 and R2 were universally high, being above 55 for camptothecin and for 10-hydroxy-camptothecin (Table 2, group I). In contrast, estimations of the R4 ratio showed that when comparing two yeasts expressing similar levels of hTOP1, a difference in RAD52 expression did not alter significantly the sensitivity of the yeast to camptothecin, with R4 values of 0.9 and 1.3. Conversely, the R3 ratio was not evaluable in the absence of any growth inhibition of yeast transformants Y(–/+) and Y(+/+) at the highest concentrations tested (Table 2, group I). Overall, 10-hydroxy-camptothecin had a similar pattern of differential sensitivities to that of camptothecin, except for its lower potency.

### Identification of RAD52-associated DNA-damaging agents

When evaluating seven anti-tumour agents with known DNA-damaging properties, namely amsacrine, etoposide, mitoxantrone, TOP 53, doxorubicin, cisplatin and bleomycin (DeVita et al, 1993; Utsugi et al, 1996), yeast transformants Y(+/–) and Y(–/–) proved to be hypersensitive, whereas yeast Y(–/+) and Y(+/+) showed diminished sensitivity or complete resistance at the concentrations tested, as revealed by the $IC_{50}$ values obtained (Table 2). Full data for etoposide and cisplatin are illustrated in Figure 1C and 1D. Based on these similarities in their patterns of differential sensitivities, these compounds were placed in group II (Table 2). More specifically, first, the influence of the RAD52 status on the sensitivity of a pair of yeasts challenged with these compounds, irrespective of their TOP1 status, was evaluated through calculation of the ratio of the $IC_{50}$ values obtained with two yeasts differing only in terms of their RAD52 expression, i.e. either yeast transformants Y(–/–) and Y(–/+) that are both devoid of TOP1 expression, or yeast Y(+/–) and Y(+/+) that both express hTOP1. In this way the R3 and R4 ratios may be considered. Amsacrine, etoposide, mitoxantrone, TOP 53, doxorubicin, cisplatin and bleomycin all yielded R3 and R4 ratio values well below unity, indicating that the integrity of RAD52 is a strong prerequisite for the resistance of yeast to these compounds (Table 2, group II). Interestingly, it appeared that the cytotoxicity generated by amsacrine, etoposide, mitoxantrone and TOP 53 was strictly dependent upon the absence of the RAD52 protein, as exemplified by the curves of growth inhibition presented for example, for etoposide (Figure 1C). Doxorubicin, cisplatin and bleomycin, while also exhibiting RAD52-dependent cytotoxicity, at their lower concentrations, totally inhibited the growth of the four yeast

© 1999 Cancer Research Campaign 

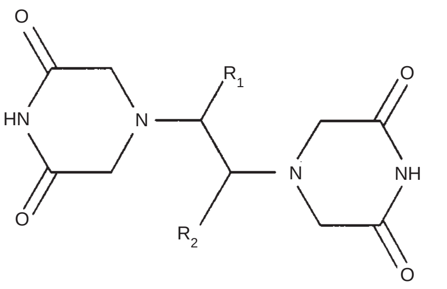

**Figure 2** Structure of bisdioxopiperazine ICRF compounds. ICRF-159: $R_1$ = H, $R_2$ = $CH_3$ (racemic); ICRF-186: $R_1$ = H, $R_2$ = $CH_3$ ((S)–(–)); ICRF-187: $R_1$ = H, $R_2$ = $CH_3$ ((S)–(+)); ICRF-193: $R_1$ = $R_2$ = $CH_3$ (meso); ICRF-201: $R_1$ = $R_2$ = $C_2H_5$ (meso); ICRF-202: $R_1$ = $CH_3$, $R_2$ = $C_2H_5$ (erythro)

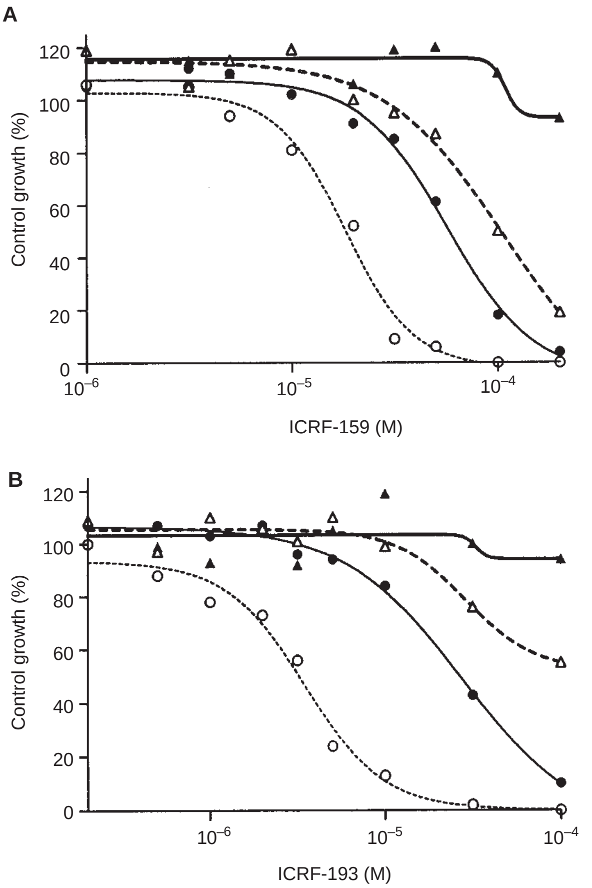

**Figure 3** Curves of growth inhibition associated with two bisdioxopiperazine compounds, ICRF-159 (**A**) and ICRF-193 (**B**). Yeast transformants studied were Y(+/–) : (Δ ----); Y(–/–) : (○ ----); Y(–/+) : (● —); Y(+/+): (▲ —). Data were plotted using a Windows-based Prism program included in this functional assay when assayed at the higher range of concentrations, as exemplified by the curves of growth inhibition induced by, for example, cisplatin (Figure 1D). Secondly, the significance of the TOP1 status on the sensitivity of yeast in the absence of any RAD52-associated variation, could be assessed through an examination of the two ratios R1 and R2. In this respect, these ratios either approximated to unity or were not evaluable due to a general lack of cytotoxicity, indicating that the TOP1 level did not influence drug sensitivities.

Finally, vinorelbine, a tubulin-targeting antitumour agent (Johnson et al, 1996) was assayed as an example of a drug with no known interaction with either DNA or with TOP1. This compound did not alter the growth of any of the four yeasts at concentrations ≤ 100 μM (data not shown). As a further control, a standard antifungal agent, the antibiotic fungizone, was shown to inhibit the growth of all four yeast recombinants with similar efficiencies (data not shown). Finally, the possibility was considered that DMSO, known for its potentially antioxidant properties, might antagonize the action of certain DNA damaging agents included in this study, e.g. cisplatin or bleomycin. Assays were performed in parallel with these test compounds solubilized in their preferred solvent. Irrespective of the solvent used, comparable results were obtained (data not shown).

### Analysis of six bisdioxopiperazine derivatives

Bisdioxopiperazine compounds are catalytic inhibitors of TOP2 (Andoh and Ishida, 1998). ICRF-187 (dexrazoxane) is the (+)-(S)-enantiomer and ICRF-186 the (–)-(R)-enantiomer of the racemic ICRF-159 [(+/–)-1,2-bis(3,5-dioxopiperazin-1-yl)propane], while ICRF-193, ICRF-202 and ICRF-201 are corresponding butane and hexane derivatives (Figure 2) which possess greater potency in mammalian cell culture and TOP2 inhibition assays (Hasinoff et al, 1995). These six bisdioxopiperazine derivatives were assayed at concentrations ranging from $10^{-4}$ to $10^{-7}$ M. Compounds ICRF-159, ICRF-202, ICRF-186 and ICRF-193 induced similar, yet distinctive, patterns of differential growth inhibition and were therefore included as group III test compounds (Table 2). ICRF-159 (Figure 3A) proved overall least cytotoxic of these four compounds with $IC_{50}$ values ranging from 18 to 100 μM and ICRF-193 (Figure 3B) was most potent with $IC_{50}$ values as low as 3.5 μM for yeast transformant Y(–/–), excluding yeast Y(+/+). Indeed, the yeast transformant Y(+/+), expressing both hTOP1 and RAD52, appeared to show either only slight sensitivity to ICRF-159, ICRF-202 and ICRF-186, or none at all to ICRF-193. Examining the individual responses of the four yeast transformants to each of these ICRF compounds in terms of their ratios of $IC_{50}$ values (Table 2), it is apparent that: (i) dual deficiencies in both TOP1 and RAD52, as in yeast Y(–/–), resulted in transformants showing maximal levels of sensitivities to these compounds; (ii) the loss of a functional RAD52 protein in yeast Y(+/–) or the absence of the TOP1 enzyme in yeast Y(–/+), provided transformants with intermediate sensitivities to these compounds; (iii) the expression of both hTOP1 and RAD52 in yeast Y(+/+) provided transformants showing, at the highest concentrations tested, either only slight sensitivity or no sensitivity at all to these compounds. The two other compounds evaluated in this series, ICRF-187 and ICRF-201 appeared far less cytotoxic than ICRF-159 (Table 2, group III). They notably impaired only the growth of yeast lacking TOP1 and a functional RAD52, i.e. yeast transformant Y(–/–). Therefore, in terms of overall relative effects, the order of increasing potency was revealed as follows: ICRF-187 < ICRF-201 < ICRF-159 < ICRF-186 < ICRF-202 < ICRF-193.

### DISCUSSION

A series of standard antitumour agents tested in the described functional assay in yeast were correctly identified according to their known mechanism(s) of action, and therefore classified as follows: (group I) drugs specifically inhibiting TOP1, such as

*© 1999 Cancer Research Campaign*

camptothecin and 10-hydroxycamptothecin; and (group II) drugs altering the integrity of double-stranded DNA. This latter group includes a number of apparently unrelated agents, although alterations in double-stranded DNA have generally been implicated in their varied mechanism(s) of action, either including interstrand DNA adduct formation, as with cisplatin, or via double-stranded DNA breaks as with bleomycin, or through a direct interference with the activity of TOP2, as with etoposide, amsacrine, mitoxantrone, TOP 53 and doxorubicin.

Furthermore, our findings show that etoposide, amsacrine, mitoxantrone and TOP 53, whose known mode of action involves stabilization of cleavable complexes formed by DNA and TOP2, exert a negative effect on yeast growth only when the RAD52 locus is disrupted, i.e. on yeast recombinants Y(–/–) and Y(+/–), but not on Y(–/+) and Y(+/+). This indicates that this set of test compounds has a mode of action appearing mainly to rely on TOP2-mediated induction of double-stranded DNA breaks repairable by the RAD52-associated DNA repair mechanism. In contrast, the mode(s) of action of bleomycin, cisplatin and doxorubicin leading to growth inhibition appeared to be only partially RAD52-dependent, since a restoration of a normal level of RAD52 protein only decreased the level of growth impairment by less than tenfold (Table 2, ratios R3 and R4). This suggests that besides this alteration of double-stranded DNA integrity, bleomycin, cisplatin and doxorubicin may actually cause alternative DNA damage such as that already known to be formed by a number of chemotherapeutic compounds, for example monofunctional or bifunctional adduct formation, intercalation of DNA thereby modifying the topology of DNA, or single- or double-strand breaks repaired by RAD52-independent epistasis groups, inhibition of other vital enzymes, generation of toxic free radicals, etc. (Thielmann et al, 1993; Abe et al, 1994; van Rosmalen et al, 1995; Chaney and Sancar, 1996). However, this hypothesis is only speculative, since the lack of sensitivity of yeast to etoposide, amsacrine, mitoxantrone and TOP 53 may actually originate from a lack of sensitivity within the ranges of concentrations tested. Alternatively, the sensitivity of RAD52-undisrupted yeast to high concentrations of bleomycin, cisplatin and doxorubicin could result from a hypothetical saturation of the RAD52 pathway. In terms of TOP1 inhibition, other groups have demonstrated that in the presence of wild-type level of yTOP1, the disruption of RAD52 was associated with a higher level of sensitivity of the recipient yeast to camptothecin (Eng et al, 1988; Nitiss and Wang, 1988). This apparent discrepancy may be explained, at least in part, by three non-exclusive hypotheses: firstly, experimental protocols essentially differed in terms of the time factor involved (ranging from several hours to several days) which may modify the overall number of cells sensitive to camptothecin whose killing has an S phase dependency. Secondly, our results may suggest a complete inactivation, by stochiometric saturation, of the RAD52 pathways by hTOP1, but not by yTOP1, in the presence of camptothecin. Thus no differences in cytotoxicity are observed when RAD52 is intact or knocked out. Thirdly, the unique genetic backgrounds inherent to each yeast strain used in this and other studies may actually differ in terms of, still uncharacterized, repair pathways that may actually compensate for the lack of RAD52 protein, notably in yeast recombinants Y(+/–). Further work will be needed to evaluate the role of other RAD52 epistasis group components as well as alternative repair mechanisms. Overall, this functional assay in yeast has served readily to identify such drugs as TOP1 inhibitors and/or agents that alter the integrity of double-stranded DNA. Notably, this functional assay has revealed that the presence of either hTOP1 enzyme and/or a functional RAD52-dependent double-stranded DNA repair mechanism may circumvent the known effect(s) of bisdioxopiperazine compounds on the activity of yeast TOP2 expressed, undisrupted, in yeast. In the case of ICRF-193, both restoration of a functional RAD52 protein and the expression of hTOP1 helped to restore cell growth to near normal levels, i.e. increased the $IC_{50}$ value by two logs (Figure 3). This apparently unique pattern of differential sensitivities essentially differed from that of all the other antitumour agents tested (Table 2) and yet was common to all the bisdioxopiperazine derivatives tested. Additionally, as shown in Figure 1C, an absence of etoposide-induced differential sensitivity between yeast transformants Y(+/–) and Y(–/–) indicated that the plasmid-driven variation in TOP1 expression in these yeast recombinants was not substituted by a concomitant alteration in yeast TOP2 levels.

The RAD52-related hypersensitivity to ICRF compounds of yeast transformants Y(–/–) and Y(–/+) appear to confirm, although using a different methodology, data reported earlier by Ishida et al (1995) describing the differential cytotoxicity exerted by ICRF-159 on the yeast strains SAR and SAR52. Furthermore, there appears relative concordance, between the order of cytotoxic potency identified using this yeast functional assay and earlier reported data from mammalian cell culture assays (Hasinoff et al, 1995), with ICRF-202 and ICRF-193 proving the most potent and ICRF-187 and ICRF-159 the least potent derivatives. The relatively lower activity exerted by ICRF-187 versus its enantiomer ICRF-186, unlike earlier results from mammalian systems (Hasinoff et al, 1995), may be indicative of differential species-specific bio-availability or enzymatic processing/inactivation of ICRF compounds. Notably, it is possible that one isomer is more sensitive to hydrolysis (causing inactivation of TOP2 activity) by the dihydropyrimidine aminohydrolase (DHPase) present in yeast, since it has been shown that bovine liver DHPase enzyme can hydrolyse one ring of ICRF-187, although effects on ICRF-186 were not investigated (Hasinoff et al, 1991).

Bisdioxopiperazine compounds are known to be catalytic inhibitors of TOP2 exerting their effects at a late stage of the catalytic cycle of the enzyme, neither causing DNA breaks nor intercalating DNA (Sehested et al, 1993; Sehested and Jensen, 1996). In these respects they differ from other TOP2 inhibitors that stabilize cleavable complexes, such as etoposide or amsacrine (Corbett and Osheroff, 1993). Indeed, bisdioxopiperazines are considered to induce DNA-bound TOP2 enzyme into a closed-clamp form that consequently triggers the further processing of DNA, notably chromosomal condensation and decondensation (Roca et al, 1994). Based on the novel results presented here, a revised mechanism of action of these bisdioxopiperazine ICRF compounds can be proposed tentatively. It can be concluded that ICRF compound-induced DNA-TOP2 complexes lead, directly or indirectly, to DNA lesions recognized by the RAD52-associated repair pathway. In addition, it appears that the absence of the TOP1 enzyme is associated with increased sensitivity to these bisdioxopiperazines. Indeed, if TOP1 is dispensable in yeast, its absence in cells is compensated by the activity of TOP2 enzyme. However, this leads to a TOP2 enzyme expressing functions and acting on DNA at sites that are not its ‘normal’ targets. One may therefore hypothesize that, within this in vivo cellular model, bisdioxopiperazines compounds essentially differ from the other TOP2-interacting compounds tested in that they preferentially target these ‘illegitimate’ functions of the TOP2

© 1999 Cancer Research Campaign enzyme that are implicated as a result of down-regulation of the TOP1 enzyme.

These bisdioxopiperazines therefore provide examples of antitumour drugs with pleiotropic mechanisms of action and of potential therapeutic importance, since, in addition to their known TOP2-mediated interactions, they appear able preferentially to impair the growth of yeast with altered DNA repair mechanisms (Chaney and Sancar, 1996), and/or decreased TOP1 expression, a phenotype implicated in resistance to TOP1 inhibitors (Pinedo et al, 1997).

In conclusion, the set of isogenic yeast recombinant developed in this study allows for the unequivocal discrimination of hTOP1 inhibitors inducing single-stranded DNA breaks, i.e. whose actions are not reparable by RAD52-dependent mechanisms, from TOP2 inhibitors and other double-stranded DNA breaking agents. Furthermore, this functional assay in yeast has proved useful in permitting a detailed dissection of the mechanism of action of a series of bisdioxopiperazine ICRF compounds, providing the first direct evidence that the cytotoxic activity of TOP2-inhibiting bisdioxopiperazine derivatives is associated with double-stranded DNA breaks recoverable by the RAD52-dependent epistasis group, and is modulated by the activity of the TOP1 enzyme. Bisdioxopiperazines therefore possess an apparent mode of action quite distinctive from other known TOP2 inhibitors evaluated.

### ACKNOWLEDGEMENTS

We are indebted to Dr J Nitiss (St Jude Children's Hospital, USA) for providing yeast strains and plasmid constructs. We thank Drs JL Nitiss and IA Hickson for helpful discussions during the preparation of this manuscript. We would also like to thank Anne Limouzy for her technical contribution and Marian Cabailh for her secretarial skill. AMC thanks the British Technology Group for support.

### REFERENCES

Abe H, Wada M, Kohno K and Kuwano M (1994) Altered drug sensitivities to anticancer agents in radiation-sensitive DNA repair deficient yeast mutants. *Anticancer Res* **14**: 1807–1810

Andoh T and Ishida R (1998) Catalytic inhibitors of DNA topoisomerase II. *Biochim Biophys Acta* **1400**: 155–171

Ausubel FM, Brent R, Kingston RE, More DD, Seidman JG, Smith JA and Struhl K (1995) *Current Protocols in Molecular Biology*. Current Protocols: Boston

Barret J-M and Hill BT (1998) DNA repair mechanisms associated with cellular resistance to antitumor drugs: potential novel targets. *Anticancer Drugs* **9**: 105–123

Bennett CB, Snipe JR and Resnick MA (1997) Persistent double-strand break destabilizes human DNA in yeast and can lead to $G_2$ arrest and lethality. *Cancer Res* **57**: 1970–1980

Bjornsti MA, Benedetti P, Viglianti GA and Wang JC (1989) Expression of human DNA topoisomerase I in yeast cells lacking yeast DNA topoisomerase I: restoration of sensitivity to the antitumor drug camptothecin. *Cancer Res* **49**: 6318–6323

Chaney SG and Sancar A (1996) DNA repair, enzymatic mechanisms and relevance to drug response. *J Natl Cancer Inst* **88**: 1346–1360

Chen AY and Liu LF (1994) DNA topoisomerases: essential enzymes and lethal targets. *Annu Rev Pharmacol Toxicol* **34**: 191–218

Corbett AH and Osheroff N (1993) When good enzymes go bad: conversion of topoisomerase II to a cellular toxin by antineoplasic drugs. *Chem Res Toxicol* **6**: 585–597

Creighton AM (1971) Piperazine derivatives. UK Patent 1234935.

Creighton AM (1974) 3,5-Dioxopiperazine derivatives. UK Patent 1374979.

DeVita VT Jr, Hellman S and Rosenberg SA (1993) *Cancer, Principles and Practice of Oncology*, 4th edn. JB Lippincott, Philadelphia

Eng WK, Faucette L, Johnson RK and Sternglanz R (1988) Evidence that DNA topoisomerase I is necessary for the cytotoxic effects of camptothecin. *Mol Pharmacol* **34**: 755–760

Friedberg EC, Siede W and Cooper AJ (1991) Responses to DNA damage in yeast. In: *The Molecular and Cellular Biology of the Yeast Saccharomyces*, Broach JR, Pringle JR and Jones EW (eds), pp. 147–192, Cold Spring Harbor Laboratory Press: Cold Spring Harbor

Hasinoff BB, Kushchak TI, Yalowich JC and Creighton AM (1995) A qsar study comparing the cytotoxicity and DNA topoisomerase II inhibitory effects of bisdioxopiperazine analogs of ICRF-187 (dexrazoxane). *Biochem Pharmacol* **50**: 953–958

Hasinoff BB, Reinders FX and Clarck V (1991) The enzymatic hydrolysis-activation of the adriamycin cardioprotective agent (+)-1,2-bis(3,5-dioxopiperazinyl-1-yl)propane. *Drug Metab Disposition* **19**: 74–80

Hays SL, Firmenich AA and Berg P (1995) Complex formation in yeast double-strand breaks repair: participation of rad51, RAD52, rad55, and rad57 proteins. *Proc Natl Acad Sci USA* **92**: 6925–6929

Ishida R, Hamatake M, Wasserman RA, Nitiss JL, Wang JC and Andoh T (1995) DNA topoisomerase II is the molecular target of bisdioxopiperazine derivatives ICRF-159 and ICRF-193 in *Saccharomyces cerevisiae*. *Cancer Res* **55**: 2299–2303

Johnson SA, Harper P, Hortobagyi GN and Pouillart P (1996) Vinorelbine: an overview. *Cancer Treat Rev* **22**: 127–142

Kingsbury WD, Boehm JC, Jakas DR, Holden KG, Hecht SM, Gallagher G, Caranfa MJ, McCabe FL, Faucette LF, Johnson RK and Hertzberg RP (1991) Synthesis of water-soluble (aminoalkyl) camptothecin analogues: inhibition of topoisomerase I and antitumor activity. *J Med Chem* **34**: 98–107

Milne GT and Weaver DT (1993) Dominant negative alleles of RAD52 reveals a DNA repair/recombination complex including Rad51 and RAD52. *Genes Dev* **7**: 1755–1765

Nitiss J and Wang JC (1988) DNA topoisomerase-targeting antitumor drugs can be studied in yeast. *Proc Natl Acad Sci USA* **85**: 7501–7505

Osheroff N (1989) Biochemical basis for the interactions of type I and type II topoisomerases with DNA. *Pharmac Ther* **41**: 223–241

Pinedo HM, Longo DL and Chabner BA (1997) *Cancer Chemotherapy and Biological Response Modifiers Annual 17*. Elsevier: Amsterdam

Pommier Y, Tanizawa A and Kohn KW (1994) Mechanisms of topoisomerase I inhibition by anticancer drugs. *Adv Pharmacol* **29B**: 73–92

Ramotar D and Masson JY (1996) *Saccharomyces cerevisiae* DNA repair processes: an update. *Mol Cell Biochem* **158**: 65–75

Roca J, Ishida R, Berger JM, Andoh T and Wang JC (1994) Antitumor bisdioxopiperazines inhibit yeast DNA topoisomerase II by trapping the enzyme in the form of a closed protein clamp. *Proc Natl Acad Sci USA* **91**: 1781–1785

Sehested M and Jensen PB (1996) Mapping of DNA topoisomerase II poisons (etoposide, clerocidin) and catalytic inhibitors (aclarubicin, ICRF-187) to four distinct steps in the topoisomerase II catalytic cycle. *Biochem Pharmacol* **51**: 879–886

Sehested M, Buhl P, Sorensen BS, Holm B, Friche E and Demant EJF (1993) Antagonistic effect of the cardioprotector (+)-1,2-bis(3,5-dioxopiperazinyl-1-yl)propane (ICRF-187) on DNA breaks and cytotoxicity induced by the topoisomerase II directed drugs daunorubicin and etoposide (VP-16). *Biochem Pharmacol* **46**: 389–393

Shen DW, Akiyama SI, Schoenlein P, Pastan I and Gottesman MM (1995) Characterisation of high-level cisplatin-resistant cell lines established from cross-resistance and protein changes. *Br J Cancer* **71**: 676–683

Subramanian D, Rosenstein BS and Muller MT (1998) Ultraviolet-induced DNA damage stimulates topoisomerase I–DNA complex formation in vivo: possible relationship with DNA repair. *Cancer Res* **58**: 976–984

Thielmann HW, Popanda O, Gersbach H and Gilberg F (1993) Various inhibitors of DNA topoisomerases diminish repair-specific DNA incision in UV-irradiated human fibroblasts. *Carcinogenesis* **14**: 2341–2351

Utsugi T, Shibata J, Sugimoto Y, Aoyagi K, Wierzba K, Kobunai T, Terada T, Oh-Hara T, Tsuro T and Yamada Y (1996) Antitumor activity of a novel podophyllotoxin derivative (TOP-53) against lung cancer and lung metastatic cancer. *Cancer Res* **56**: 2809–2814

van Hille B and Hill BT (1998) Yeast cells expressing differential levels of human or yeast DNA topoisomerase II: a potent tool for identification and characterisation of topoisomerase II-targeting antitumor agents. *Cancer Chemother Pharmacol* **42**: 345–356

*© 1999 Cancer Research Campaign*

Van Rosmalen A, Cullinane C, Cutts SM and Phillips Don R (1995) Stability of adriamycin-induced DNA adducts and intrastrand crosslinks. *Nucleic Acids Res* **23**: 42–50
Wall ME, Wani MC, Cooke CE, Palmer KH, McPhail AT and Slim GA (1966) Plant antitumor agents. I. The isolation and structure of camptothecin, a novel alkaloidal leukemia and tumor inhibitor from *Camptotheca acuminata*. *J Am Chem Soc* **88**: 3888–3890
Wang JC (1985) DNA topoisomerases. *Annu Rev Biochem* **54**: 665–697
Wood RD (1996) DNA repair in eukaryotes. *Annu Rev Biochem* **65**: 135–167

© 1999 Cancer Research Campaign 
